# Biopreservation and Bioactivation Juice from Waste Broccoli with *Lactiplantibacillus plantarum*

**DOI:** 10.3390/molecules28124594

**Published:** 2023-06-07

**Authors:** Patryk Zdziobek, Grzegorz Stefan Jodłowski, Edyta Aneta Strzelec

**Affiliations:** Department of Fuels Technology, Faculty of Energy and Fuels, AGH University of Krakow, 30-059 Kraków, Poland; jodlowsk@agh.edu.pl (G.S.J.); strzelec@agh.edu.pl (E.A.S.)

**Keywords:** biotransformation, biopreservation, *Lactiplantibacillus plantarum*, polyphenols

## Abstract

The content of polyphenols, lactic acid, and antioxidant properties in fermented juice increases more at 30 °C than at 35 °C during the lactic fermentation process in butanol extract and broccoli juice. The concentration of polyphenols is expressed by phenolic acid equivalents as gallic acid-Total Phenolic Content (TPC), ferulic acid (CFA), p-cumaric acid (CPA), sinapic acid (CSA), and caffeic acid (CCA). The polyphenols present in fermented juice exhibit antioxidant properties and the ability to reduce free radicals using total antioxidant capacity (TAC) assay, while also the percentage of the DPPH (2,2-Diphenyl-1-picrylhydrazyl) radical and ABTS (2,2′-azino-bis (3-ethylbenzothiazoline-6-sulfonic acid) cation radical scavenging activity. Lactic acid concentration (LAC), total flavonoid content as quercetin equivalents (QC), and acidity increases during the work of *Lactiplantibacillus plantarum* (previously *Lactobacillus plantarum*) in broccoli juice. The pH was monitored during the process of fermentation in both temperatures (30 °C and 35 °C). Densitometric measurements of lactic bacteria (LAB) showed increasing concentration at 30 °C and 35 °C after 100 h (~4 h), but the value concentration dropped after 196 h. The Gram staining showed only Gram-positive bacilli *Lactobacillus plantarum* ATCC 8014. The Fourier transform infrared (FTIR) spectrum for the fermented juice showed the characteristic carbon–nitrogen vibrations that may originate from glucosinolates or isothiocyanates. Among the fermentation gases, more CO_2_ was released from fermenters at 35 °C than at 30 °C. The biopreservation used *Lactiplantibacillus plantarum* to prevent the problem of food waste of plant origin. The probiotic bacteria used in fermentation have a very beneficial effect on health and the human body.

## 1. Introduction

The current taxonomy uses the name *Lactiplantibacillus plantarum* and we use this name in the paper; however, the purchase of the strain from the ATCC collection was made under the name *Lactobacillus plantarum*. Therefore, the previous name was included in the summary and every section in the article related to ATCC collection.

The aim of the conducted research was to check the possibility of increasing the content of antioxidants and their further use in technological processes or the production of dietary supplements [1]. The process of food preservation with the use of *Lactiplantibacillus plantarum* was chosen because the literature reports other LAB species did not provide a satisfactory increase in polyphenols [2]. In previously unpublished research by the authors focusing on cruciferous plants as commonly preserved by lactic fermentation showed that broccoli produced the highest concentration of polyphenols. In the end, we decided to use fresh broccoli processing waste.

The popularity of fermented products has increased in recent years. It is also associated with the growing social awareness of healthy eating and the emergence of diseases. Broccoli (*Brassica oleracea* var. *Italica Plenck*) is a green cruciferous vegetable belonging to the cabbage family. Originally, broccoli arose from the leaves and flowers of wild cabbage (*Brassica oleracea*) [3]. Both florets, leaves, and the stalks of broccoli contain glucosinolates, isothiocyanates, vitamins, phenolic compounds, minerals, and fiber. Broccoli is a plant with beneficial properties, namely, anti-inflammatory, anti-cancer neuroprotective, and renoprotective effects. Clinical research shows that broccoli can be eaten by humans at any age. Various parts of broccoli can be used after processing as a functional food with high health promotion potential [4,5]. The concentration of total phenolic acid after 8 days of fermentation increased (30.23 ± 5.31 mg/mL) compared with the first day of fermentation (4.16 ± 0.04 mg/mL). The main phenolic acids that can be found in broccoli are caffeic acid, chlorogenic acid, gallic acid, ferulic acid, and sinapic acid [6]. During fermentation processes with the use of the *Lactiplantibacillus* strain, reduced forms of phenolic acids are produced. Fermented broccoli juice has much more bioavailable and bioactive forms of phenolic acids [7]. Lactic fermentation of broccoli is an alternative to products made from broccoli without the use of heat. In addition, it also favors the biotransformation of glucosinolates to isothiocyanates [8]. *Lactiplantibacillus plantarum* is found in fermented food, in the digestive tract, and is used in the food industry as a probiotic starter culture. These strains have been reported to particularly reduce the risk of cardiovascular disease, while also significantly inducing mucosal, humoral, and cellular immune responses, reducing kidney stones, and producing varied concentrations of exopolysaccharide with anticancer properties [9]. Rajoka et al. reported that they obtained exopolysaccharide from *Lactiplantibacillus plantarum.* An exopolysaccharide showed excellent techno-functional properties, potent immunomodulatory activity in vitro, and increased immunomodulatory activity in immunosuppressed mice. *Lactiplantibacillus plantarum* MM89 strain was used for the production of probiotic fermented human breastmilk with exopolysaccharide [10]. By observing the different properties of *Lactiplantibacillus plantarum*, it can be concluded that an interesting probiotic and analytical research showed the presence of several fundamental probiotic characteristics, which can successfully colonize and inhabit the human digestive system [11]. *Lactiplantibacillus* bacteria also produce potent bacteriocins, which are antimicrobial substances capable of preserving food and acting as an antibiotic. The presence of probiotic bacteria in the human body may contribute to strengthening immunity, as well as reducing the level of bad cholesterol in the blood [12,13]. The fiber in plant material has a positive effect on the human digestive tract and plays an important role in maintaining the health and well-being of humans. The occurrence of fiber together with lactic acid bacteria in the digestive tract contributes to increasing the colony of these bacteria, which in turn stimulates the innate immune response body [14]. The construction of a recombinant *Lactiplantibacillus plantarum* strain expressing the SARS-CoV-2 spike protein can be used as a promising food-grade oral vaccine candidate against SARS-CoV-2 infection [15]. Yang et al. [16] conducted a study on the effects of aqueous fermented broccoli on the chronic inflammation of the gastrointestinal tract, including gastritis, ulcers, and gastric tumors induction by the *Helicobacter pylori* bacteria. They showed that the presence of *Lactiplantibacillus plantarum* bacilli inhibits the growth of *Helicobacter* bacteria. The fermented broccoli using the *Lactiplantibacillus plantarum* MG208 strain may constitute a functional diet for the treatment of *Helicobacter pylori* infections [16]. Mousavi et al. [17] confirmed that the rate of glucose transformation is faster than fructose to lactic acid transformation by *Lactiplantibacillus plantarum*. Due to the large amount of wasted food in the world, it is worth preventing it from being thrown away and preparing probiotic functional food in the form of fermented vegetables and juices. When processing broccoli for food purposes, the inflorescences are mainly used, while the stalks-heads are usually cut off even at the stage of sale on the market. These residues can be used for fermentation purposes. Polyphenols can also be used in food processing, thus influencing the physicochemical properties of the substance, stabilizing the flavor and texture, or preserving the food itself. Moreover, using nanoencapsulation, polyphenolic compounds can be protected against the negative influence of pH and oxidizing factors, thereby enhancing the effect of active ingredients of functional food during human nutrition. It must be remembered that many polyphenols are responsible for the aroma and stabilization of the color of the fruit. They can play this role in food processing [18]. It was also noticed that the addition of polyphenols to dairy products not only increases their functional properties, but also increases their sensory values [19]. In the publication [20], the authors show the possible use of apple peel polyphenols as a strawberry preservative. A similar approach was presented in [21,22] for the preservation of chilled meat with the usage of tea polyphenols. Another use of tea polyphenols can be their addition to noodles or pasta in order to improve their physicochemical properties, such as mechanical strength and flexibility, as well as sensory properties [23]. Polyphenols can be potentially used as a catalyst for the Fenton reaction [24] in the treatment of wastewater in an advanced oxidation process of organic compounds, including possibly chlorinated ones. A very interesting technical application of polyphenols is the ecologically friendly functionalization of drilling fluid to reduce fluid losses in the drilling process, both at room temperature and elevated temperatures, as is the case in reservoir conditions [25]. The above list is not exhaustive of all possible uses of polyphenols. It is possible to find information that in addition to many health-promoting activities, they can also be used as anti-corrosion additives, in photography or cosmetics [26]. In our opinion, polyphenols/flavonoids can be used as substitutes for synthetic antioxidants added to fuel biocomponents to prevent aging. They appear here as eco-friendly additives that replace toxic agents. However, research on this possibility is ongoing.

## 2. Results and Discussion

**Figure 1:**
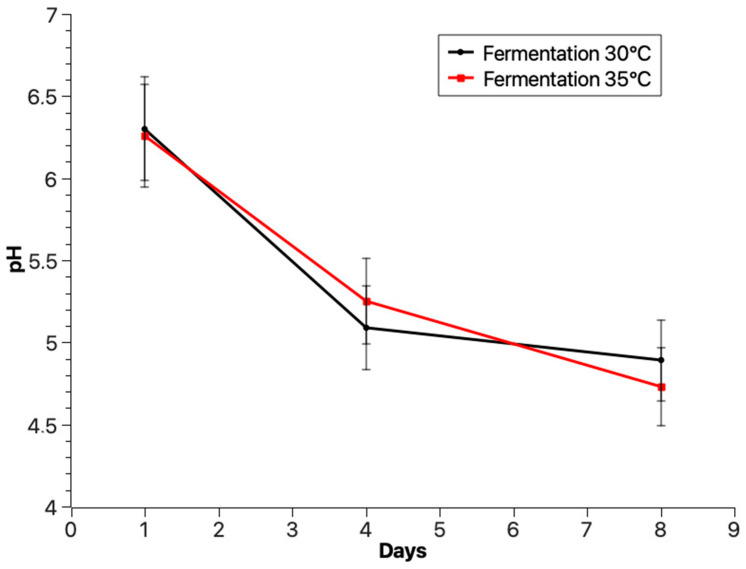
Changes in pH of fermented juices of broccoli for 8 days of fermentation at 30 °C (black) and 35 °C (red).

Parameters such as pH were measured during the fermentation of broccoli to evaluate the progress of the process. As a result, the pH change was noticed after 4 and 8 days of fermentation (Figure 1). The average pH of fermented broccoli juice after 4 days of fermentation was higher at 35 °C compared with 30 °C (Figure 1).

**Figure 2:**
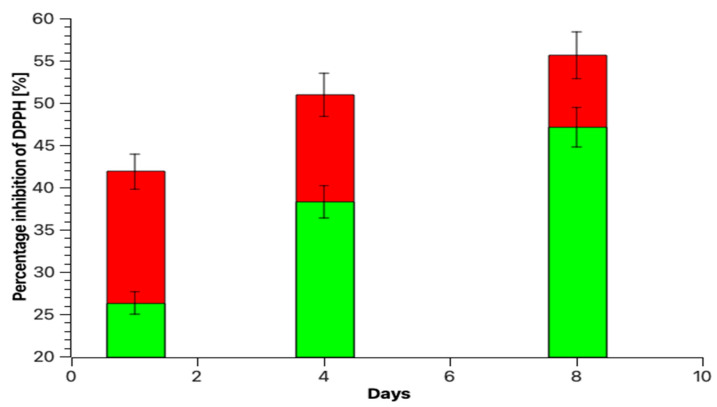
DPPH radical scavenging capacity of extracts of fermented broccoli juices at 1, 4, and 8 days at 30 °C (red) and 35 °C (green).

**Figure 3:**
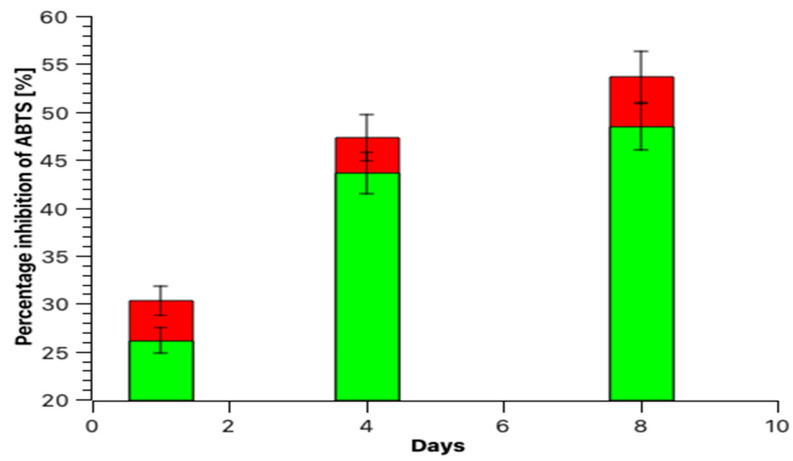
ABTS cation radical scavenging capacity of extracts of fermented broccoli juices at 1, 4, and 8 days at 30 °C (red) and 35 °C (green).

However, after 8 days of fermentation, the average pH value was lower at 35 °C compared with 30 °C. The average value of pH of brine in fermenters working at 35 °C was 4.7 ± 0.23 and in fermenters working at 30 °C was 4.96 ± 0.24 (Figure 1). During the fermentation process, the habitat of *Lactiplantibacillus plantarum* becomes more acidic. A mean pH value of 4.68 was measured by Kiczorowski et al. [27] after 7 days of fermentation of broccoli, as tested measurements on the 8th day of fermentation (Figure 1). Comparing the obtained pH results with the pH value was conducted by Major et al. [28] for sauerkraut, in which one can notice a similarity to broccoli, which, just like cabbage, belongs to cruciferous vegetables (Figure 1). Millán et al. [1] presented an initial pH of 7.85, which decreased on the third day of fermentation (4.73–4.96), followed by a second reduction on day 6 (4.20–4.30). A similar analysis conducted by Iga-Buitrón et al. [2] indicated changes in pH that also decreased from ~5.8 to 4.53 (no bacilli culture) and 3.99 (using *Levilactobacillus brevis*, *Lactococcus lactis)* on the second day of fermentation. On day 4 of fermentation, the pH values decreased significantly in fermentation spontaneously by ~4.00 and ~3.60 by *Lactobacillus* cultures. Values of pH less than ~4.60 during fermentation allowed the prevention of fermented food. The research group of Shokri et al. [29] in their report presented changes in pH by fermentation in the case of fermented broccoli floret purees from 6.8 during the first time and 3.8 during the final time. The acidity of fermented broccoli juice increases quite quickly due to the presence of lactic acid, as well as organic acids such as acetic acid, propionic acid and butanoic acid [27,30]. The influence of the time of fermentation process on the total antioxidant properties of butanol extract of fermented juice of broccoli at temperatures 30 °C and 35 °C is shown in Figure 2 and Figure 3.

**Figure 4:**
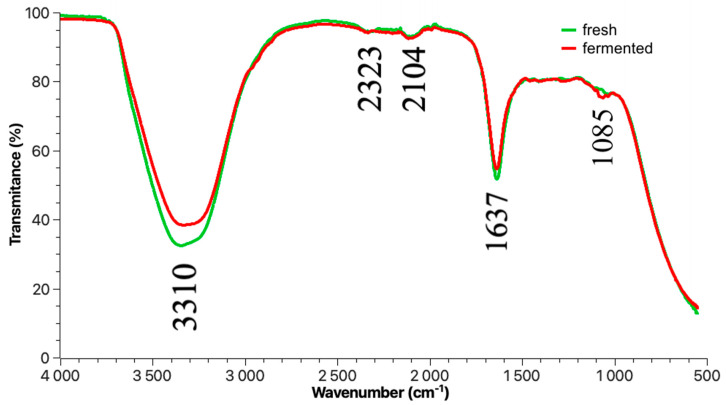
ATR-IR spectrum from the fermented and fresh juice of broccoli.

Scavenging percentage inhibition of DPPH and ABTS grows during the fermentation of broccoli at two different temperatures. The maximum inhibition of DPPH and ABTS is achieved on day 8 of fermentation. Comparing the effect of temperature on the percentage of radical and cationic radical inhibition shows that at a higher temperature, the percentage of inhibition is lower than at a lower temperature. In the first 4 days of fermentation, the percentages of inhibition of DPPH are at 30 °C (50.96 ± 2.53%) and 35 °C (38.32 ± 1.87%), and in the case of ABTS the percentage inhibition was at 30 °C (47.29 ± 2.36%) and at 35 °C (43.12 ± 2.11%). The highest degree of inhibition of radical DPPH and cation radical ABTS on the 8th day was at 35 °C and 30 °C. (Figure 2 and Figure 3). Hou et al. [31] performed studies using freeze-dried fermented juice of broccoli, after fermentation at 60 h, DPPH and ABTS radical scavenging capacity were significantly increased. The percentage inhibition of DPPH from ~56.00% (initial) increased to ~62.50% (fermentation end point) and the percentage inhibition of ABTS increased from~35.00% (initial) to ~68.00% (fermentation end point). Cai et al. [8] presented the antioxidant capacity of broccoli puree during lactic acid bacteria fermentation and storage at 25 °C and 4 °C using oxygen radical absorbance capacity (ORAC) and obtained results of the growth of antioxidant potential with time. During lactic acid fermentation, the antioxidant properties and vitamin C content increase, but this is not always the rule. This was proven by the research group of Tolonen et al. [32]. The spectra in Figure 4 shows the specific vibration of the sample fermented and fresh juice of broccoli after the 8th day of the process using *Lactiplantibacillus plantarum* bacilli.

The peaks around 3310 cm^−1^ are responsible for an -OH group. It is the characteristic functional group for liquid samples, polyphenols, and carboxylic acids. The intensity at 1637 cm^−1^ is presented by amide-stretching bands. Vibration is characteristic of ketones or carbonyl grouping located in carboxylic groups. Carbon–carbon triple bonding at 2104 cm^−1^ is popular in terminal alkanes. There is also a very narrow band in the spectra around 2323 cm^−1^, which involves the presence of C=N stretch vibration. Maybe the carbon–nitrogen bond comes from glucosinolates or isothiocyanates. Compounds are much more concentrated in cruciferous vegetables including broccoli (Figure 4) [33]. The spectra of powdered puree of fermented broccoli looks different. The characteristic peaks in the FTIR spectra of all solid samples of fermented broccoli researched by Jian-Hui Ye et al. [34] are different from the spectra using the liquid sample from the fermented juice of broccoli in Figure 4. The specific vibration band of spectra was in the range of 2800–3000 cm^−1^ signed to methyl group (–CH_3_) and the peaks at ∼1550 and ∼1640 cm^−1^ were identified as amide derivatives [34,35]. The estimated concentration of phenolic acids was presented in Table 1 as gallic acid (TPC), ferulic acid (CFA), p-cumaric acid (CPA), sinapic acid (CSA), and caffeic acid (CCA) per mg/100 mL. The content of phenolics compounds increases during the 8 days of fermentation (Table 1). The greatest concentration in Table 1 among phenolic acids after the 8th day of fermentation has (TPC) gallic acid at 30 °C (30.22 ± 4.6 mg/100 mL); P-coumaric acid (CPA) has the lowest content (26.89 ± 3.47 mg/100 mL) in the tested extracts of fermented broccoli juice after 8 days of fermentation. TPC, CFA, CPA, CSA, and CCA (phenolic acids) content in Table 1 is higher at 30 °C than at 35 °C. Higher total polyphenol (TPC) content when using other *lactobacillus* strains (*Levilactobacillus brevis* and *Lactococcus lactis*) and spontaneous fermentation using by 10 days were found by Iga-Buitrón et al. [2], who reported from the initial day to the tenth day of fermentation (23.88–23.72 mg/g) by spontaneous fermentation and by starter cultures (24.90–23.72 mg/g). In that work, the total concentration of polyphenols remains constant at approx. 24 mg/g. In the case of our research, the content of total polyphenols (TPCs), CFA, CPA, CSA, and CCA increased during the entire time of fermentation. Perhaps an excessively high temperature (35 °C) deactivates the lactic acid bacteria, and as a result of biotransformation fewer polyphenols are transferred to fermented juice (Table 1). The content of lactic acid (LAC) and total flavonoid content (QC) was presented in Table 2. Similarly, the content of TPC, CFA, CPA, CSA, and CCA and the content of flavonoids as quercetin equivalents (QC) are also higher at 30 °C than at 35 °C. Salas-Millán et al. [1], using the method LC-ESI-QqQ-MS/MS, reported the concentration of metabolites of phenolic compounds by spontaneous fermentation of broccoli stalks without dressing or dressed with garlic: chlorogenic acid (CHL) ~0.64 mg/L, sinapoyl hexose (SNP-Hex) ~0.82 mg/L, 4-*O*-feruloyl quinic acid (4FQa) ~0.65 mg/L, quercetin-3-O-diglucoside (QdG) ~0.17 mg/L, sinapic acid (SNP) ~0.18 mg/L, kaempferol 3-O-diglucoside (KdG) ~0.06 mg/L, rosmarinic acid (RosA) ~1.06 mg/L, 1,2-disinapoyl-gentiobiose (dSNPGb) ~0.16 mg/L and 1-sinapoyl-2-feruloyl-gentibiose (SnFrGb) ~0.20 mg/L with maximum concentration by three days of fermentation. Likewise, Iga-Buitrón et al. [2] identified the use of analysis of derivative phenolic acids and flavonoids in fermented juice of broccoli. Among those identified, mainly phenolic acids and flavonoids, are: 3-Caffeoylquinic acid (Family of Hydroxycinnamic acids), Quercetin 3-*O*-xylosyl-glucuronide and Quercetin 3-*O*-galactoside (Family of Flavonols), Caffeic acid 4-*O*-glucoside and p-Coumaric acid 4-*O*-glucoside (Family of Hydroxycinnamic acids), Ferulic acid 4-*O*-glucoside (Family of Methoxycinnamic acids). In this work, the concentration of phenolic acids and flavonoids was estimated and expressed using equivalents. The results showed the differences in conducting fermentation at two different temperatures. The lactic acid concentration (LAC) increased after 8 days of fermentation both at 30 °C from ~11.00 to ~46.00 and at 35 °C from ~9.00 to ~28.00 mg/100 mL. Iga-Buitrón et al. [2] reported the total acidity was significantly higher in (cultures *Lactobacillus* fermentation) i-FB (13.26 ± 0.5 g/L) than in (spontaneous fermentation) s-FB (11.85 ± 0.5 g/L), which was expressed as lactic acid equivalents. The results obtained in this research showed that at higher temperatures than the room temperature, a higher content of lactic acid was received.

**Figure 5:**
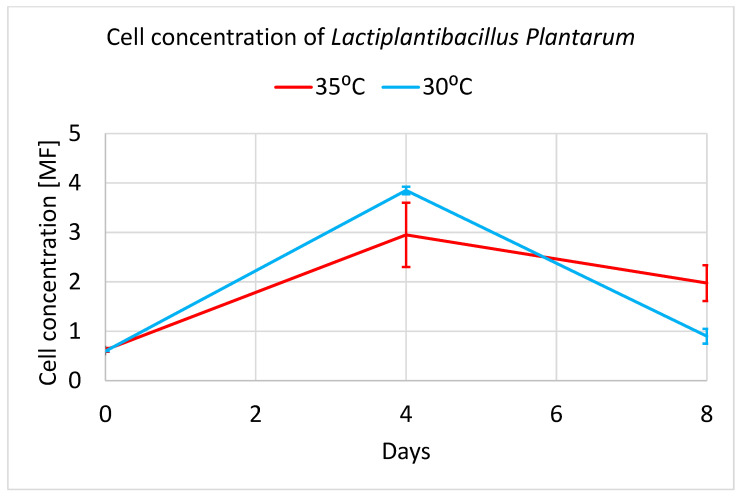
Influence of 30 °C and 35 °C temperatures and concentration on growth in fermentation with *L. plantarum* [MF] depending on the time.

Flavonoids (mainly flavanols) are among the most popular polyphenolic compounds found in cruciferous vegetables. Likewise, phenolic acids, including hydroxycinnamic acids, occur in various species of *Brassica* [8]. The lactic acid bacteria used for fermentation in this experiment was *Lactiplantibacillus plantarum*. Two fermentation temperatures were selected, namely 30 °C and 35 °C. Based on the graphs in Figure 5, it can be clearly stated that both temperatures are optimal for the growth of *L. plantarum*.

However, when fermenting for 196 h (8 days), the difference in bacterial growth can be seen. At the temperature of 30 °C, the growth of bacteria is fast and reaches a maximum on day 4 and decreases quickly. At 35 °C, bacterial growth is slower and reaches a maximum on day 4 and slowly decreases, but not as low as in the case of 30 °C. Interestingly, bioreactors operating at a temperature of 35 °C were more differentiated in terms of bacterial concentration. This was shown in the higher standard deviations of the 35 °C averages. In the analysis group Shokri et al. [29] presented the maximum growth of cells with broccoli puree fermented after 2 days using mixed culture (*Leuconostoc mesenteroides* (BF1 and BF2; 1:1) and *Lactiplantibacillus plantarum* (B1). Salas-Millán et al. [1] noted the supreme level of growth of lactic acid bacteria (LAB) after three times by beginning the spontaneous fermentation of broccoli. In our case analysis, a similar maximum growth of *Lactobacillus plantarum* ATCC 8014 was reported on the fourth day of fermentation both at 30 °C and 35 °C temperatures. It is likely the use of temperatures above room temperature hinders bacterial growth a little.

**Figure 6:**
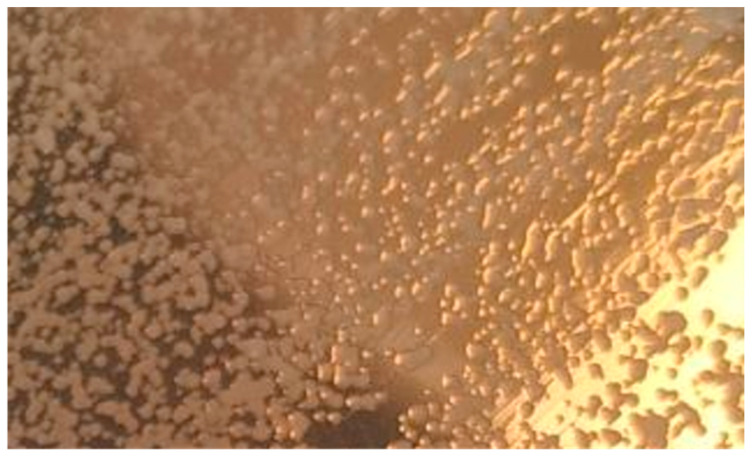
*Lactobiplanticillus plantarum* after 48 h of incubation in a laboratory incubator at 35 °C.

**Figure 7:**
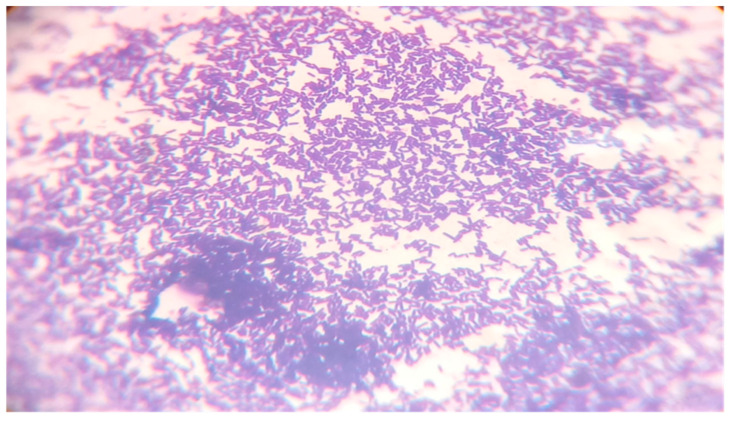
*Lactobacillus plantarum* ATCC 8014 under the microscope after Gram’s staining. If bacteria stay purple, they are Gram-positive. If the bacteria stay red or pink, they are Gram-negative.

In both cases (30 °C and 35 °C), after about ~100 h, the maximum number of bacteria occurs, as originally assumed when planning the experiment. As a result, the study of the number of bacteria showed that the *Lactiplantibacillus plantarum* bacteria copes very well with fermentation at a slightly elevated temperature. To confirm the presence of the *Lactiplantibacillus plantarum* bacteria in pure culture in the medium, a qualitative analysis was performed consisting of a microscopic analysis of the obtained bacterial cultures. A positive organoleptic result allowed us to state that the bacteria obtained in the culture are pure cultures of *Lactiplantibacillus plantarum* which is presented in Figure 6. The *Lactiplantibacillus plantarum* starter culture for bioreactor inoculation was cultivated on a sterilized Man, Rogosa, and Sharpe (MRS). A medium from a commercially obtained ATCC 8014 sample. Bacteriocins (lactoline) are produced by *Lactiplantibaccillus plantarum* to stop the growth of other familiar Gram+ and Gram- microorganisms [36], so there was no expected growth of other strains of *Lactiplantibacillu* ssp. Bacteria that display purple stains when exposed to crystal violet are referred to as Gram-positive bacteria. While those that are stained red with carbol fuchsin are called Gram-negative bacteria [37]. Figure 7 shows the violet staining of the *Lactobacillus plantarum* ATCC 8014 bacteria involved in the lactic fermentation of broccoli. The random samples gained from bioreactors and stained purple bacteria indicate that they belong to Gram-positive bacteria [37,38].

The Gram staining method was used not for the identification of bacteria, but for the confirmation of the specified growth. The test provided in this investigation contains the collection of three samples for every fermenter taken randomly from the substrate and every identified sample does not contain any other microorganism. Figure 7 shows an example of a microscopic image of one such sample.

As hetero fermentation was carried out with *Lactiplantibacillus* ssp. in addition to the main metabolite, which is lactic acid, it also provides carbon dioxide, thus the measurement of the volume and composition of the fermentation gas can be used to monitor the fermentation progress on an ongoing basis [39,40]. The measurement of the volume of gas produced during lactic fermentation and the study of its composition leads to conclusions about the course of the process.

**Figure 8:**
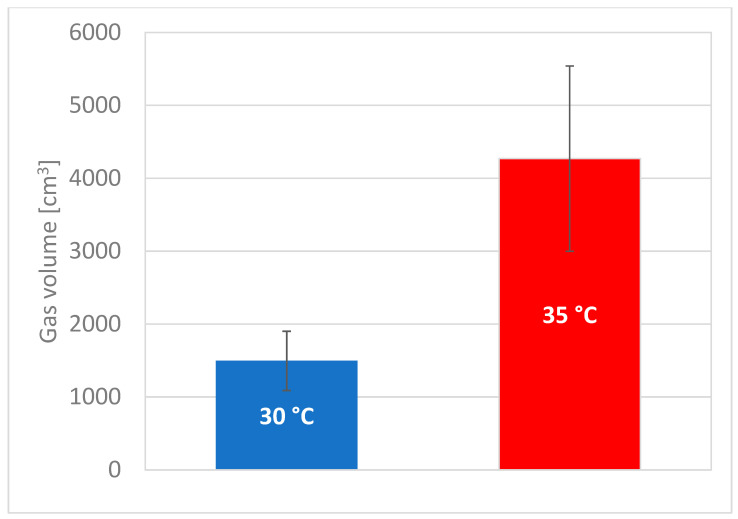
The cumulative volume of produced fermentation gas.

**Figure 9:**
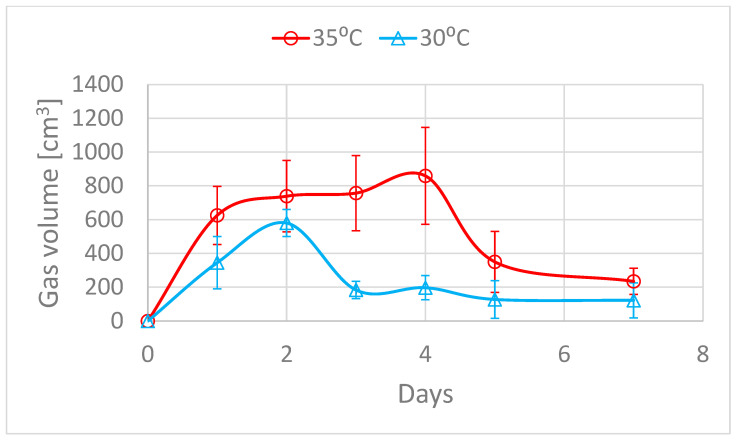
Changes in the production of gas during the fermentation.

Fermenters were thermally conditioned in two groups at 30 and 35 °C. The higher operating temperature of the fermenter generally resulted in higher volumes of fermentation gas, even up to 3 times more. (Figure 8). There was a significant decrease in the volume of gas produced after 4 days in almost every fermenter (Figure 9).

This marks the end of the turbulent fermentation period, during which the greatest increase in bacterial metabolic products is achieved and a peak in bacteria growth. The data from the gas composition analysis is interesting, as there are also significant differences between the two temperatures with this parameter. The content of selected fermentation gas components is presented in Table 3.

At the temperature of 35 °C, the average CO_2_ content in the fermentation gas is higher than at the temperature of 30 °C, and it increases noticeably (Table 3). On the other hand, at a temperature of 30 °C, the average CO_2_ concentration in the gas increases slightly. Fermenters operating at lower temperatures differ more in this parameter (bigger SD). This is confirmed by the well-known fact that a temperature of 35 °C is more suitable for this bacterium. An even more interesting property of the fermentation gas is the change in the content of H_2_S within 5 days. In the case of fermenters operated at 30 °C, its content is lower and decreases over time, while at 35 °C the concentration of H_2_S is higher and increases, with the highest measured H_2_S content (581 ppm) for the bioreactor with the highest fermentation gas production. This means that a higher temperature is also suitable for putrefying bacteria, which can partially impair the function of the lactic acid bacteria.

A likely explanation for this situation is the LAB metabolic pathway. We suspect that at higher temperatures, the metabolic pathway favors CO_2_ and lactic acid at lower temperatures. The tests were carried out on two sets of four fermenters simultaneously, and all showed the same trends. Similarly, polyphenol content increases more at lower temperatures during lactic fermentation.

## 3. Materials and Methods

### 3.1. Chemicals and Reagents

Butanol and sodium chloride (NaCl) were obtained from Avantor (Gliwice, Poland). Sodium carbonate, 80% lactic acid, ferric chloride hexahydrate (FeCl_3_·6H_2_O) and potassium persulfate (K_2_S_2_O_8_) (pure for analysis) were obtained from Chempur (Gliwice, Poland). Gallic acid, ferulic acid, p-coumaric acid, sinapic acid, and caffeic acid and quercetin (HPLC grade) were purchased from Acros Organics (Geel, Belgium). Folin–Ciocalteu reagent, 2,2′-Azino-bis (3-ethylbenzothiazoline-6- sulfonic acid) diammonium salt (ABTS), Gram′s crystal violet solution, and 2,2-Diphenyl-1- picrylhydrazyl (DPPH) were obtained from Sigma Aldrich (St. Louis, MO, USA).

### 3.2. Material

Plant Material Broccoli with the ending use-by date was obtained from a local farmers’ market in the old town of Cracow (Poland).

### 3.3. Inoculation, Fermentation, and Sampling

*Lactobacillus plantarum* ATCC 8014 (American Type Culture Collection (ATCC) were inoculated on Petri dishes on an agar medium (MRS LAB-AGAR TM, Biomaxima S.A., Lublin, Poland) intended for the cultivation of *Lactiplantibacillus* strains. The nutrient solution was prepared following the instructions on the package. The sown bacteria were incubated for 48 h in a laboratory incubator at 35 °C (CLN 53STD, POL-EKO Aparatura, Poland). After incubation, 1 mL of *Lactiplantibacillus plantarum* bacteria was transferred to 5 mL of liquid medium and incubated for 24 h in a laboratory incubator in bacteriological tubes. Before use, the lab tubes were sterilized in the flame of the burner each time.

Broccoli florets were grated for homogenized material. Broccoli was used for fermentation with a use-by date that has ended in order to highlight the problem of throwing out food and to show how to use it. Subsequently, the prepared substrate (400 g) was transferred into eight bioreactors (1000 mL) and 550 mL of water was poured into each of them. Each of the four bioreactors set was left standing using a water bath at 30 °C and 35 °C. Samples of fermented juices were taken after the 1-, 4-, and 8-day fermentation process.

### 3.4. Physicochemical Measurements, Processes and Analysis

#### 3.4.1. pH Measurements

During the fermentation, the pH was measured after the first, fourth, and eighth day of the lactic acid fermentation process at 30 °C and 35 °C of each bioreactor of juice. Before taking measurements, the pH meter was calibrated each time with the buffers 4.00, 7.00, and 9.00 pH. The results were obtained using a portable pH meter (Elmetron CPC-105, Zabrze, Poland).

#### 3.4.2. Extraction

Before the measurements of polyphenols, butanol/fermented juice (1:1) extractions were carried out using 2 mL tubes at 6000 [rpm] for 15 min on an HTL mini centrifuge (Corning HTL, Warsaw, Poland). The butanol extract was taken for measurements. Butanol is a solvent that has little miscibility with water and is therefore ideally suited for the extraction of fermented juices.

#### 3.4.3. Sterilization

Before starting the fermentation, the bioreactors with the capacity of 1000 mL with plastic caps were sterilized for 15 min using an autoclave (Yeson YS-18L-E, Ningbo Yinzhou Yeson Medical Device Ningbo Co., Ningbo, China). Each of the 8 fermenters used were sterilized to prevent contamination with microorganisms and molds from the air.

#### 3.4.4. Carbon Dioxide Content

The life process of *Lactiplantibacillus* spp. is related to CO_2_ emission, so measuring its emission during fermentation makes it possible to control the ongoing process without additional interference. The gas was collected independently from several fermenters into gas burettes filled with brine. The brine acted as an indicator of the gas volume in the gas burette, at the same time the use of a saturated NaCl solution was necessary to reduce the solubility of CO_2_ in water. The gas composition was controlled by a GA5000 (GeoTech, Great Britain) automatic analyzer with FTIR indications for CH_4_ and CO_2_ and electrochemical cells for O_2_, H_2_, and H_2_S.

### 3.5. Phytochemical and Microbial Measurements and Analysis

#### 3.5.1. Polyphenols Content

The polyphenols content was determined using the Folin–Ciocalteu assay described by Ainsworth et al. [41]. Compactly, 0.1 mL of the sample butanol extract was mixed with 0.5 mL 2M Folin–Ciocalteu reagent and 6 mL distilled water. After 3 min, 1.5 mL of saturated sodium carbonate was added and up to 10 mL of distilled water was poured in. The reaction was incubated at 40 °C for 30 min and the absorbance was read at 760 nm (Metash Model UV-5600, Shanghai, China). The total phenolic content (TPC) was standardized against gallic acid and expressed as mg of gallic acid equivalents per 100 mL of the sample. The results were calculated against a standard curve of gallic acid (y = 3.7867x − 0.2144; serial dilutions of gallic acid, 0.1, 0.2, 0.3, 0.4, 0.5, 0.6, 0.7, 0.8, 0.9, and 1.0 mg/mL; coefficient of determination, R^2^ = 0.991) and expressed as mg GAE/100 mL. Additionally, the derivatives of cinnamic acid were investigated. Serial dilutions of ferulic acid, p-coumaric acid, sinapic acid, and caffeic acid, 0.1, 0.2, 0.3, 0.4, 0.5, 0.6, 0.7, 0.8, 0.9, and 1.0 mg/mL. The content of ferulic acid (CFA) was expressed as mg of ferulic acid per 100 mL of sample. The results were calculated against a standard curve of ferulic acid (y = 0.7048x + 0.2144; coefficient of determination, R^2^ = 0.991) and expressed as mg FAE/100 mL. The content of p-cumaric acid (CPA) was expressed as mg of p-cumaric acid per 100 mL of sample. The standard curve of p-coumaric acid (y = 0.5668x + 0.0299; coefficient of determination, R^2^ = 0.984) and expressed as mg CAE/100 mL. The content of sinapic acid (CSA) was expressed as mg of sinapic acid per 100 mL of the sample. The standard curve of caffeic acid (y = 0.5423x + 0.0265; coefficient of determination, R^2^ = 0.994) was expressed as mg SAE/100 mL. The content of caffeic acid (CCA) was expressed as mg of caffeic acid per 100 mL of sample. The standard curve of caffeic acid (y = 0.8913x + 0.0136; coefficient of determination, R^2^ = 0.990) was expressed as mg CFE/100 mL. The volume of the mixture was prepared for 10 mL completed with distilled water.

#### 3.5.2. Total Flavonoid Content

The total flavonoid content (TFC) was estimated using the aluminum chloride colorimetric assay described by Phuyal et al. [42]. Briefly, 1.0 mL of the sample was mixed with 4 mL of distilled water. At the same time, 0.3 mL of 5% NaNO_2_, and 0.3 mL of 10% AlCl_3_ after 5 min. Then, 2 mL of 1 M NaOH was added to the mixture after 6 min. The volume of the mixture was prepared for 10 mL completed with distilled water. The content of quercetin (CQ) was expressed as mg of quercetin per 100 mL of the sample. The results were calculated against a standard curve of quercetin (y = 1.2279x − 0.0865; serial dilutions of quercetin, 0.1, 0.2, 0.3, 0.4, 0.5, 0.6, 0.7, 0.8, 0.9, and 1.0 mg/mL; coefficient of determination, R^2^ = 0.999) and expressed as mg QE/100 mL. The absorbance of samples was read at 510 nm using a spectrophotometer (Metash Model UV-5600, Shanghai, China).

#### 3.5.3. Lactic Acid Content

The lactic acid concentration (LAC) was determined by means of a modified spectrophotometric method using FeCl_3_·6H_2_O as a reagent as described by Borshchevskaya et al. [43]. Before the measurements, 2 mL of juice samples were centrifuged using an ultracentrifuge. The supernatant was used for further analysis. Then, 50 µL of fresh fermented juice broccoli was mixed with 2 mL 0.2% FeCl_3_· 6H2Osolution. The reaction mixture was incubated at 25 °C and was shaken for 8 min using an orbital shaker (RSLAB-7, Beriáin, Spain). After incubation, the absorbance was read at 390 nm using a spectrophotometer (Metash Model UV-5600, Shanghai, China). LAC was standardized against 80% lactic acid and expressed as mg of lactic acid equivalents per 100 mL sample of fermented juice. The results were calculated against a standard curve of lactic acid (y = 0.015x − 0.0099; serial dilutions of lactic acid, 2, 4, 6, 8, 10 mg/mL; coefficient of determination, R^2^ = 0.993) and expressed as mg LA/100 mL.

#### 3.5.4. Total Antioxidant Capacity

The determination of the free radical scavenging activity of butanol extracts from fermented broccoli juices was carried out using two complementary methods, namely, DPPH and ABTS as described by Abramovič et al. [44]. For the first assay, 2.9 mL of 40 mg/mL DPPH solution in methanol was mixed with 0.1 mL of butanoic extracts and was incubated at 30 min at room temperature. Subsequently, the absorbances of the samples were measured at 515 nm using a spectrophotometer (Metash Model UV-5600, Shanghai, China). For the second assay, ABTS^•^+ radical cation was formed by the reaction of 7 mmol/L of ABTS diammonium salt and 2.45 mmol/L of K_2_S_2_O_8_. The mixture was allowed to stand for 12 h at room temperature in a dark green solution. For the purpose of measurement, 1 mL of this ABTS solution was mixed with 10 µL of butanoic extracts and was measured in terms of absorbance at 734 nm after 30 min incubation (Metash Model UV-5600, Shanghai, China). The analysis was performed as described by Kusznierewicz et al. [45].

Percentage of inhibition (%) = (ABScontrol − ABSsample)/ABScontrol × 100.

ABS control—The absorbance of ABTS or DPPH radical solutions.

ABS sample—The absorbance of a sample with DPPH or ABTS after 30 min incubation.

#### 3.5.5. Optical Density of Lactiplantibacillus Bacteria Growth

Bacterial density was tested using a densitometer DEN-1 (bioSan, Riga, Latvia), a device capable of measuring the turbidity of the solution in McFarland units. The densitometer is used to measure the cell concentration (bacterial cells, yeast) during fermentation in order to determine the sensitivity of microorganisms to antibiotics, as well as to identify microorganisms in various tests to measure the optical density at a specific wavelength, and to determine the concentration of the colored solution absorbing green light. The principle of operation is based on the measurement of optical density with a digital presentation of results in McFarland units [MF]. One McFarland roughly corresponds to a density of 3 × 10^8^ CFU/cm^3^ of *Escherichia coli* strain ATCC25922 (Colony Forming Units (CFUs)). This is a very convenient form of determining the number of bacteria in liquids in microbiological analysis in this study. Photos under a microscope were taken using Gram′s crystal violet solution at microscope Genetic Pro Bino (Delta Optical, Mińsk Mazowiecki, Poland).

#### 3.5.6. FT-IR Spectroscopy

Spectra after the 8th day of fermentation of broccoli juice were run on FTIR Perkin Elmer Frontier spectrometer (Perkin Elmer, Waltham, MA, USA) with an ATR detector for liquid samples. Fourier transform infrared (FTIR) spectroscopy was used to record IR absorption spectra. The FTIR spectra were measured in the wavenumber range 500–4000 cm^−1^ with 64 scans per spectrum. The FTIR tests were performed at room temperature by applying a 50 μL sample of fermented juice and fresh juice filtered through a PTFE microfilter 0.45 μm on the crystal of the Attenuated Total Reflectance (ATR) detector.

### 3.6. Statistical Analysis

In each measurement, where possible, the standard research approach with at least three replicates of measurement on separate samples was used. The mean of such measurement and the standard deviation of the mean was determined. The exceptions were the measurements of the volume of gas from fermenters and the measurements of the optical density of bacterial cultures. In these cases, the collection of three samples was not possible or would disturb the operation of the bioreactors. Finally, the results were combined based on measurements from 8 reactors (mean and standard deviation), 4 in two groups for temperatures of 30 °C and 35 °C. In this way, tables and graphs containing data for two temperatures along with standard deviations were prepared.

## 4. Conclusions

The research demonstrates that the temperature selection influences antioxidant properties and the content of phenolic acids, lactic acid, and total flavonoid content. A higher temperature also contributes to the release of more CO_2_ among the fermentation gases. One can expect that a temperature of 35 °C is the best for lactic acid bacteria and measurements of fermentation gas containing CO_2_ prove that. However, data acquired in these investigations reveal that a lower temperature of 30 °C is good enough from the point of view of antioxidant production. The inhibition of DPPH or ABTS is on a similar level for samples at both temperatures. The population size of *Lactiplantibacillus plantarum* bacteria is also maximally on a similar level for both temperatures. Finally, it seems the lower temperature is a better choice for increasing the concentration of flavonoids (polyphenols) in liquid extracts. A lower temperature means lower costs of antioxidant production. Moreover, the production of fermentation gases, including the increased content of H_2_S, is greater at 35 °C. In summary, the process conducted at 30 °C is eco-friendly. The time of fermentation between 5 and 8 days is optimal. The measurements of gas production and evaluation of the *Lactiplantibacillus* population size show an increase up to 100 h (~4 days) and further falls, while the content of polyphenols in liquid extracts continuously increases until the 8th day of fermentation. The pH value was going down during the entire period of fermentation and the minimum value measured on the 8th day.

The FTIR spectra of fresh broccoli juice and fermented juice (effluent) are almost the same. They have the same absorbance peaks, but with different intensities. The exception is the peak at 1085 cm^−1^, where a small peak appears, probably responsible for the presence of ether or oxy groups [46]. This proves a slight deviation from the composition of raw juice. However, other measurements indicate an increase in the content of lactic acid and polyphenols.

Lactic fermentation cooperates with the preservation of food and prevents food waste. Simultaneously, that process can be used for the biotransformation of food waste or expired food to produce valuable products containing antioxidants usable as dietary supplements or add-ons for biochemical treatment in many different technology processes.

## Figures and Tables

**Table 1:** Estimation changes in TPC, CFA, CPA, CSA, and CCA of fermentation at 30 °C and 35 °C.

	Content of Phenolic Acids (mg/100 mL)				
	TPC	CFA	CPA	CSA	CCA
**30 °C**					
Day 1	4.17 ± 0.04	nd	nd	nd	nd
Day 4	24.0 ± 4.36	20.04 ± 1.7	20.74 ± 1.55	20.30 ± 2.92	20.66 ± 0.9
Day 8	30.22 ± 4.6	25.71 ±4.68	26.89 ± 3.47	27.68 ± 4.6	27.16 ± 3.33
**35 °C**					
Day 1	3.88 ± 0.14	1.94 ± 0.45	1.61 ± 0.22	1.73 ± 0.44	1.65 ± 0.25
Day 4	18.70 ± 0.87	20.14 ± 3.75	20.75 ± 5.17	18.15 ± 0.95	17.98 ± 0.78
Day 8	21.88 ± 2.29	18.59 ± 2.71	18.94 ± 2.65	21.55 ± 6.43	18.99 ± 2.36

**Table 2:** Estimation changes in QC and LAC of fermentation at 30 °C and 35°C.

Content (mg/100 mL)				
	30 °C		35 °C	
	QC	LAC	QC	LAC
Day 1	3.02 ± 0.28	11.39 ± 0.47	1.26 ± 0.09	8.86 ± 0.033
Day 4	7.73 ± 0.37	28.74 ± 0.53	6.7 ± 0.25	18.47 ± 0.88
Day 8	13.9 ± 1.48	45.56 ± 6.78	10.81 ± 1.49	27.96 ± 1.22

**Table 3:** Components of fermentation gases.

	30 °C		35 °C	
Component	Day 1	Day 4	Day 1	Day 4
CO_2_ [%]	51.35 ± 8.65	55.2 ± 6.8	74.48 ± 2.94	86.87 ± 2.84
H_2_S [ppm]	54 ± 6	15.5 ± 2.5	81 ± 7.50	390 ± 175

## Data Availability

Not applicable.
